# Compressible unsteady steam flow and heat transport analysis: a numerical investigation

**DOI:** 10.1038/s41598-022-23020-3

**Published:** 2022-10-29

**Authors:** Azad Hussain, Muhammad Arsaln, Aysha Rehman, Fahad M. Alharbi, Nevzat Akkurt, Sayed M. Eldin, Saad Althobaiti

**Affiliations:** 1 Department of Mathematics, University of Gujrat, Gujrat, 50700 Pakistan; 2 Department of Mathematics, Al-Qunfudah University College, Umm Al-Qura University, Mecca, Saudi Arabia; 3 Rare Earth Elements Application and Research Center, Munzur University, 62000 Tunceli, Turkey; 4 Center of Research, Faculty of Engineering, Future University in Egypt, New Cairo, 11835 Egypt; 5 Department of Sciences and Technology, Ranyah University Collage, Taif University, P.O. Box 11099, Taif, 21944 Saudi Arabia

**Keywords:** Computational science, Fluid dynamics

## Abstract

The unsteady compressible steam laminar flow associated with heat transfer in fluids in a squared cylinder is examined in this work. The current challenge was created utilizing the CFD approach. The laminar flow is chosen with a low Mach number. With the geometric wall, the flow has a no-slip condition. The pressure on the flow is kept at 0 pas, and the temperature in the flow regime is 305.13. A 0.5 m/s velocity is used to start the flow. With the use of graphics, the effects of time on velocity and pressure distributions are discussed. Different outcomes are also mentioned, such as drag coefficients, lift coefficients, and heat distributions. The velocity drops from 2.5 to 1.6 m/s at t = 7 s in the absence of anybody's force and temperature 305.13 K. Pressure increases from 0.00098 to 0.001 Pas in the flow interval of 10 s. Surface temperature increases from 360 to 375 K in time intervals of 10 s keeping pressure constant. And contour temperature increases from 371.56 to 374.2 K in time intervals of 10 s keeping the pressure constant. This information provides us with caution about the emission of steam from the chimneys of furnaces. It implies that when steam flows from a cylindrical geometry like chimneys of furnaces it heats the upper inner and outer parts which may destroy the material. So for safety, that emission should be taken for a short interval of time otherwise it will result in a havoc process. The lift coefficient remains constant and the drag coefficient increases from 0.0005 to 0.065. Under that condition, fluid has to face more resistance. To overcome that difficulty fluid should be provided with high velocity to continue it for a long time. The technique used to solve modeled problems is the Backward Difference Formula.

## Introduction

A smooth straightforward flow, which does not show any zigzagging is called laminar flow. Small Reynolds number possesses laminar flow. Higher Reynolds numbers, cause a transition to turbulence. Usually, the critical Reynolds number is known to be around 200.

Schubauer and Skramstad^1^ confirmed the theory, putting an end to a long-running debate and shedding new light on the causes of the transition from laminar to turbulent flow. The Navier–Stokes equations were solved by Abraham et al.^2^ for a complete description of fluid flow in a channel with a rectangular cross-section and two equally porous walls. A practical aerodynamically and structurally reasonable efficient laminar flow control (LFC) suction method is developed and then applied to a second F94 LFC wing glove in flight, removing the slowest boundary layer particles through many closed spaced fine slots: Up to the F94 test limit, 100 percent laminar flow is observed by Pfeminger et al.^3^. The history of aircraft laminar flow control (LFC) from the 1930s to the 1990s, as well as the current state of the technology, is examined by Joslin^4^. Schrauf^5^ studied natural laminar flow (NLF) and hybrid laminar flow control (HLFC). An incompressible Navier–Stokes equation developed within the priority research program is provided by Schafer et al.^6^ for 2D and 3D laminar flow.

Fluids whose density varies significantly when applied to pressure changes are dealt with by the branch of mechanics known as Compressible flow. If the flow's Mach number exceeds 0.3 then the flow is considered significantly compressible.

The principles of classical compressible flow have been used to solve problems in fields as diverse as high-speed aerodynamics and long-distance transport of low-speed gases^7^. Liu et al.^8^ Investigate the evolution of gas-vacuum interfaces for both in-viscid and viscous one-dimensional isentropic gas motions. For Upgrading the worth of time-dependent solutions of the Euler equations in two dimensions Peraire et al.^9^ described an adaptive mesh technique. Pulliam^10^ adaptive an implicit finite-difference procedure for unsteady three-dimensional flow. Artificial boundary conditions (BCs) for the simulation of inflow, outflow, and far-field (radiation) problems, with a focus on compressible turbulent shear flow techniques, are reviewed by Colonius^11^. Subgrid models for Large Eddy Simulation (LES) of compressible turbulent flow are tested by Verman et al.^12^ for the three-dimensional mixing layer.

A flow is said to be time-dependent if field variables change over time. The time-dependent study is used in electromagnetism, heat transfer, and solid mechanics.

Sung et al.^13^ presented a new scheme for unsteady networks as well as a solution algorithm based on Dijkstra's label-setting algorithm. A new technique for numerically investigating the unsteady flow of an incompressible fluid with an invidiously confined and invidiously free boundary is exposed by Harlow and Welch^14^. To generate particle traces in unsteady flow fields, a particle tracing system has been developed Lane^15^. The dispersion of a non-uniform slug in a time variable fully constructed laminar flow is authorized by Gill et al.^16^. Lolla et al.^17^ create and demonstrate the methodology for predicting the time-optimal paths of ocean vehicles in continuous dynamic flows. John^18^ describes a numerical investigation of a two-dimensional time-dependent flow around a cylinder. The Rayleigh number at which steady convective flow transitions to time-dependent flow is experimentally determined for several fluids with Prandtl numbers ranging from 1 to 10^4^ by Krishnamurti et al.^19^. Lane et al.^20^ present a method for visualizing time-dependent flow fields with streak lines. Kuangyu Shi et al.^21^ describe a method for visually analyzing the dynamic behavior of 3D time-dependent flow fields using path line behavior.

The current study reveals the numerical simulation of two-dimensional unsteady compressible laminar flow passing through a squared cylinder. Velocity and pressure distribution plots are presented for two-dimensional laminar steam flow. The results of the drag coefficient, heat distribution, and lift coefficients are also investigated. The flow has a Reynolds number of approximately150, which is sufficient to keep the flow laminar.

## Mathematical formulation

**Figure 1:**
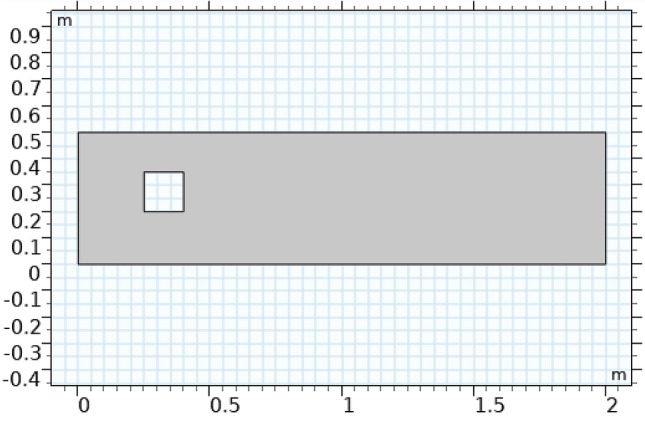
The sketch of the problem.

The region of flow is demonstrated in Fig. 1. First, the squared rectangular cylindrical geometry is constructed mathematically, whose length is 1 m, breadth is 4 m, then a square of length 0.15 m has been drawn at position (0.25 m, 0.2 m). The difference between these two figures is made and then constructed all region is highlighted. In the next stage, in the section on material properties, the properties of the fluid are added. In Fig. 1, the boundary layers possess velocity u zero. The boundary condition is considered no slip and the initial pressure kept is zero. In the inlet, a velocity of 1 m/s is given to flow to proceed and the outlet condition is selected. For solving Naiver–Stokes equations, the physics of flow is added. Figure 1 shows the boundaries of the region of flow.

The position of the rectangle and squared cylinder are as follows:

**Rectangle:** Position (0,0) Width (4 m); Height (1 m) and size (4,1).

**Square:** Position (0.25,0.2) sides (0.15).

The boundary conditions on the outer boundary are as follow:

Region ‘domain start ‘outer’ (0,0).

{mirror condition on bottom boundary}.

Natural ($$u_{r} )$$ = 0, Natural ($$u_{\theta } ) =$$ 0, value *u*_*z*_ = *0*.

Natural (p) = 0, line close to point (2,0).

No slip on sides (i.e., velocity = 0) Value *u*_*r*_ = *0*, value $u_{\theta } = 0$, u_z_ = 0.

Natural (p) = 0 line close to point (2,0.5) Natural *(u*_*r*_*)* = *0*, natural ($u_{\theta }$ = 0),

Value (u_r_) = 0, natural(p) = 0. the line to close (0,0.5).

At *t* = *0 ; u*_*r*_ = *0,* $$u_{\theta } = 0$$*, u*_*z*_ = *0, P* = *0.*

At *r* = *0.2, z* = *0.25, u* = *-U*_*o*_*n.*

The drag and lift coefficients are defined as:

$$C_{D} = \frac{{2F_{D} }}{{\rho U_{mean}^{2} D}},\quad C_{L} = \frac{{2F_{L} }}{{\rho U_{mean}^{2} D}}.$$

The equation of continuity is:

1 $$\frac{\partial p}{{\partial t}} + \nabla .\left( {\rho u} \right) = 0,$$

The equation of heat and momentum equation is:

2 $$\rho C_{p} \left( {\frac{\partial T}{{\partial t}} + u.\nabla T} \right) + \nabla .\left( {q + q_{r} } \right) = \alpha_{P} T\left( {\frac{\partial P}{{\partial t}} + u.\nabla P} \right) + \tau .\nabla u + Q$$

3 $$\rho \frac{\partial u}{{\partial t}} + \rho \left( {u.\nabla } \right)u = \nabla .\left[ { - pi + k} \right] + F,$$

4 $${\text{where }}\;K = u\left[ {\nabla u + \left( {\nabla u} \right)^{T} } \right] - \frac{2}{3}u\left( {\nabla .u} \right)u.$$

The Continuity and momentum equations will reduce to the following forms:

5 $$\frac{{\partial u_{r} }}{\partial r} + \frac{1}{r}u_{r} + \frac{{\partial u_{z} }}{\partial z} = 0,$$

6 $$\frac{{\partial u_{r } }}{\partial t} + u_{r} \frac{{\partial u_{r} }}{\partial r} + u_{z} \frac{{\partial u_{r} }}{\partial z} - \frac{{u_{\theta }^{2} }}{r} = - \frac{1}{\rho }\frac{\partial P}{{\partial r}} + \frac{4}{3}\vartheta \frac{{\partial^{2} u_{r} }}{{\partial r^{2} }} + \vartheta \left( {\frac{{\partial^{2} u_{\theta } }}{\partial r\partial z} + \frac{{\partial^{2} u_{r} }}{{\partial z^{2} }}} \right),$$

7 $$\frac{{\partial u_{\theta } }}{\partial t} + u_{r} \frac{{\partial u_{\theta } }}{\partial r} + u_{z} \frac{{\partial u_{\theta } }}{\partial z} + u_{r} u_{\theta } = \vartheta \left[ {\frac{{\partial^{2} u_{\theta } }}{{\partial z^{2} }} + \frac{{u_{\theta } }}{{r^{2} }}} \right] + \vartheta \frac{{\partial^{2} u_{\theta } }}{{\partial z^{2} }},$$

8 $$\frac{{\partial u_{z} }}{\partial t} + u_{r} \frac{{\partial u_{z} }}{\partial r} + u_{z} \frac{{\partial u_{z} }}{\partial z} = \vartheta \left[ {\frac{{\partial^{2} u_{r} }}{{\partial z^{2} }} + \frac{{\partial^{2} u_{z} }}{\partial r\partial z}} \right] + \frac{4}{3}\vartheta \frac{{\partial^{2} u_{z} }}{{\partial z^{2} }} - \frac{1}{P}\frac{\partial P}{{\partial z}}.$$

The transformations used for the heat equation are

9 $$\begin{aligned} & \alpha_{p} = - \frac{1}{\rho }\frac{\partial P}{{\partial T}},\;\sigma = - \rho I + \tau,Q_{p} = \alpha_{p} T\left[ {\frac{\partial P}{{\partial t}} + u.\nabla P} \right],Q_{vd} = \tau .\Delta u, \\ & q = - d_{m} k\Delta T,\; d_{m} = 1m^{2} .P_{r} = \frac{K}{{\rho C_{p} }},\;\eta = \frac{{z(\Omega \sin \alpha^{*} )^{0.5} }}{{V^{0.5} (1 - st\Omega \sin \alpha^{*} )^{0.5} }}, \\ & G = (\Omega \sin \alpha^{*} )^{ - 1} (1 - st\Omega \sin \alpha^{*}, \\ & T\left( {t,r,z} \right) = T = \left( {T_{\omega } - T_{\infty } } \right)\theta,T_{\omega } - T_{\infty } = \left( {T_{0} - T_{\infty } } \right)\frac{x}{L}(1 - st^{*} )^{ - 2} \\ \end{aligned}$$

After transformation we get,

10 $$\begin{aligned} & Pr(\theta^{\prime \prime } ) = \nu \left( {\frac{1}{2}\theta^{\prime } \eta + 2\theta s} \right) \\ & \quad - pr\frac{G\nu }{{k_{f} \left( {T_{w} - T_{\infty } } \right)}}\left[ {\left\{ {\frac{{q_{0} }}{{A_{s} \Delta T}}} \right\} - p\left\{ {\frac{{u_{r} }}{r} + \frac{{\partial u_{r} }}{\partial r} + \frac{{\partial u_{\theta } }}{\partial z}} \right\} - \frac{1}{3}\left\{ {\frac{{\partial u_{z} }}{\partial z}\frac{{\partial^{2} u_{\theta } }}{{\partial^{2} z}} - 3\frac{{u_{\theta } }}{r}\frac{{\partial u_{r} }}{\partial z} - \frac{{\partial^{2} u_{r} }}{{\partial^{2} z}}} \right\}} \right] \\ & \quad + pr\frac{1}{{k_{f} \left( {T_{w} - T_{\infty } } \right)}}\frac{1}{{R_{e} }}\left[ {\frac{{\partial^{3} }}{{\partial r^{3} }}\left( {\frac{4}{3}u_{r} + u_{\theta } + u_{z} } \right) + \frac{1}{3}\left( {\frac{{u_{\theta } }}{r}\frac{{\partial^{2} u_{\theta } }}{{\partial^{2} r}} + \frac{{\partial u_{r} }}{\partial z}\frac{{\partial^{2} u_{\theta } }}{{\partial^{2} r}} - \frac{{u_{\theta } }}{r}\frac{{\partial u_{\theta } }}{\partial r}} \right) + \left( {\frac{{u_{\theta } }}{r}} \right)^{2} + \frac{4}{3}\left( {\frac{{u_{r} }}{r}} \right) + \frac{4}{3}\frac{{\partial u_{\theta } }}{\partial z}} \right] \\ \end{aligned}$$

## The procedure of numerical solution

In the solution, a finer mesh controlled by physics is used. The total number of triangular elements is 25,314. The number of quadrilateral elements is 1812, the number of edge elements is 990, and the number of vertex elements is 8. The maximum size of the element is 0.014, while the minimum element size is 0.0004.

The corner refinement option is selected for domain 1. Trimming options are available for dealing with sharp corners. The laminar flow section is used to investigate this model. Table 1 describes mesh statistics. Table 2, on the other hand, describes the mesh size. The numerical solution employs a normal mesh.

**Table 1:** The thermophysical properties of steam flow are as follow.

Property	Expression	Description
Density	0.590 kg/m^3^	Density of fluid
Viscosity	0.68 pa s	The viscosity of the fluid
Temperature	32 °C	Temperature during flow

**Table 2:** Description of mesh statistics and mesh size.

Statistics	Value	Mesh	Size
Minimum quality of an element	0.4319	Geometric level of entry	Boundary
The average quality of an element	0.8433	Selection	Boundaries 2–3, 5–8
Triangular elements	4038	Calibrate for	Fluid dynamics
Quadrilateral quality of an element	0.8358	Maximum size of an element	0.067
Edge elements	330	Minimum size of an element	0.003
Vertex	8	Factor of curvature	0.4
Predefined size	Normal	Maximum growth of element rate	1.2

## Discussion and outcomes

**Figure 2:**
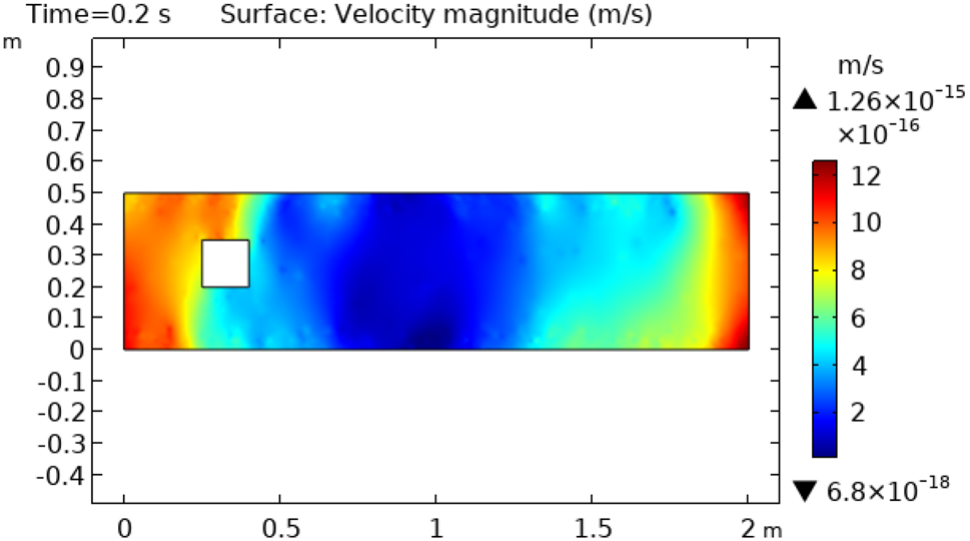
Velocity distribution in squared cylindrical flow at t = 0.2 s.

The results presented here demonstrate the laminar flow of steam in squared cylindrical geometry. The flow model has 2D geometry. The diameter of cylindrical flow is on the vertical axis, and the z direction in the current two-dimensional plots is concerned by the horizontal axis. Figure 2 represents velocity distribution at time = 2 s. This figure represents the movement of fluid that strikes a squared figured block kept in the path of flow. This figure shows that velocity varies turn by turn. The upper layers of the bottom boundary of the cylinder show peak values of velocity which is 1.2 m/s until fluid strikes the squared figure block kept in the flow of fluid. At the point of contact of fluid and squared figure block velocity decreases to a minimum value but after covering some path fluid again retains increasing values of velocities which ultimately reaches to initial velocity.

**Figure 3:**
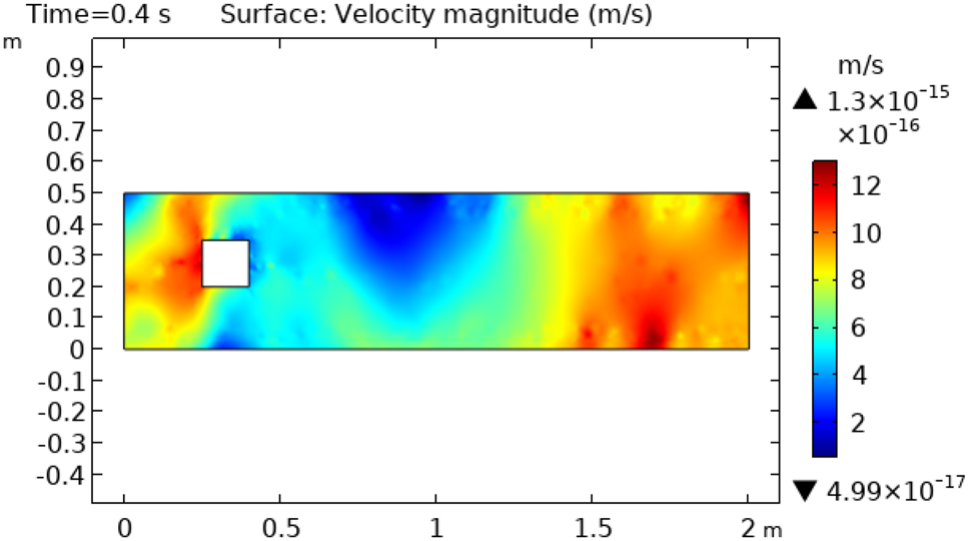
Velocity distribution in squared cylindrical flow at t = 0.4 s.

**Figure 4:**
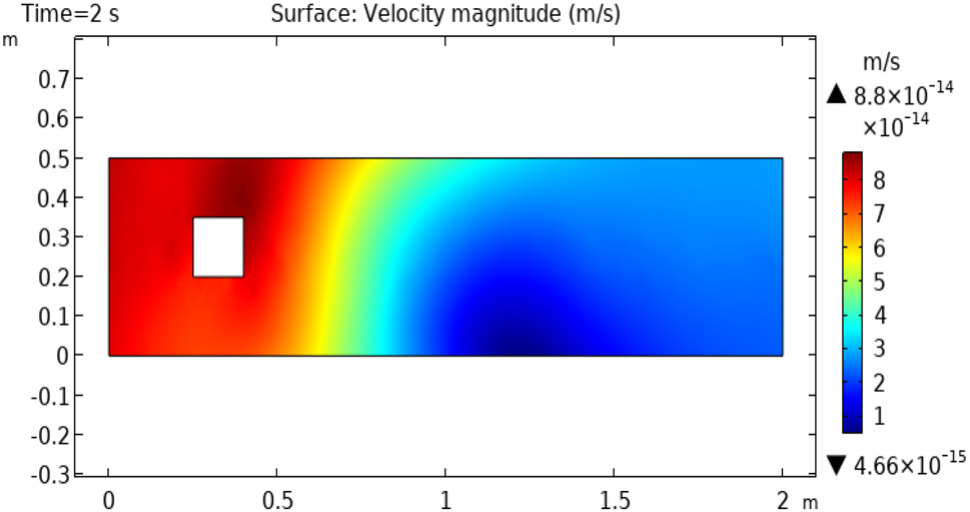
Velocity distribution in squared cylindrical flow at t = 2 s.

**Figure 5:**
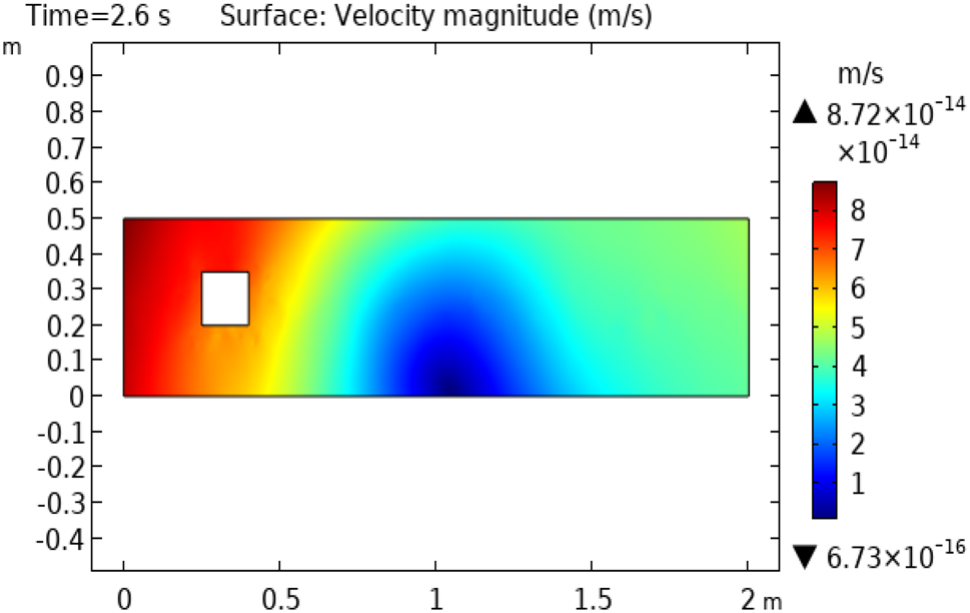
Velocity distribution in squared cylindrical flow at t = 2.6 s.

Figure 3 shows velocity distribution at time = 0.4 s. This figure describes approximately the same behavior as does Fig. 2 except for the fluid behavior which is moving away from the squared figure block. This part represents an enhanced change in velocity rather than the fluid flowing at time = 0.2 s. Figure 4 and 5 represent fluid flow at times 2 s and 2.6 s, respectively. In Fig. 4 we see that extremes of flow are changed. Now the flow starts from the initial velocity of $8.8 \times 10^{ - 16} {\text{ m}}/{\text{s}}$ and when strikes to squared cylinder this velocity reduces to $4.6 \times 10^{ - 17} {\text{ m}}/{\text{s}}.$ When the flow moves forth, the velocity increases gradually and attains its initial velocity at the extreme point of the flow. Figure 5 shows approximately the same oscillations of velocity as shown in Fig. 4. The difference is that in Fig. 5 velocity changes occur more rapidly than in Fig. 4.

**Figure 6:**
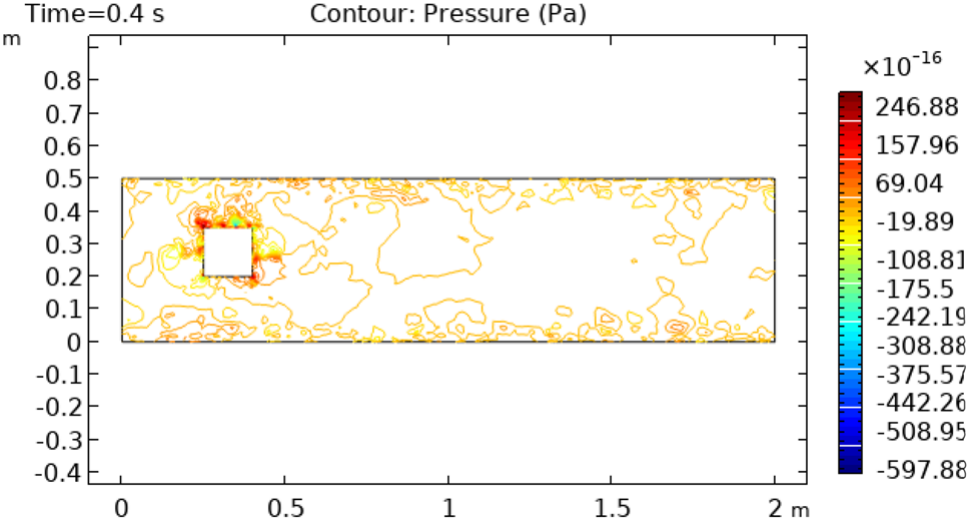
Pressure distribution in squared cylindrical flow at t = 0.4 s.

**Figure 7:**
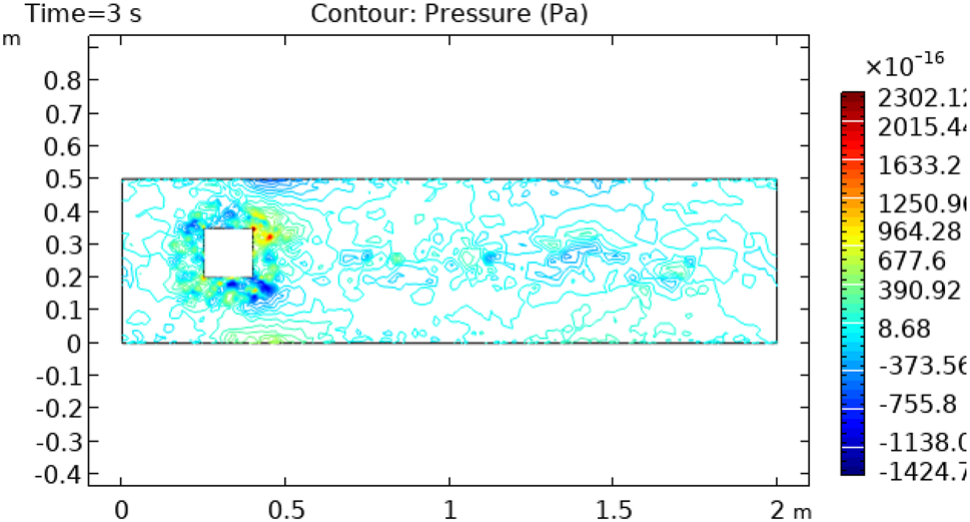
Pressure distribution in squared cylindrical flow at t = 3 s.

Figures 6 and 7 show pressure distribution around the squared figure block at time intervals of 0.2 s and 3 s, respectively. At the initial point, the pressure on the fluid is − 19.89 Pa s that increases to 646.66 Pas at the point of contact with the fluid and square block, and that pressure again reduces to its previous pressure where the fluid moves away from the block and continues with that pressure for the rest of the flow. In Fig. 7. the initial pressure on the flow is -1424.7 $\times 10^{ - 16} {\text{m}}/{\text{s}}$ Pa s which reaches 676.6 $\times 10^{ - 16} {\text{m}}/{\text{s}}$ Pa s at the connecting point of fluid and square block. When the fluid moves away from the block the pressure again reaches its initial pressure that remains unvarying up to the boundary of the cylinder.

**Figure 8:**
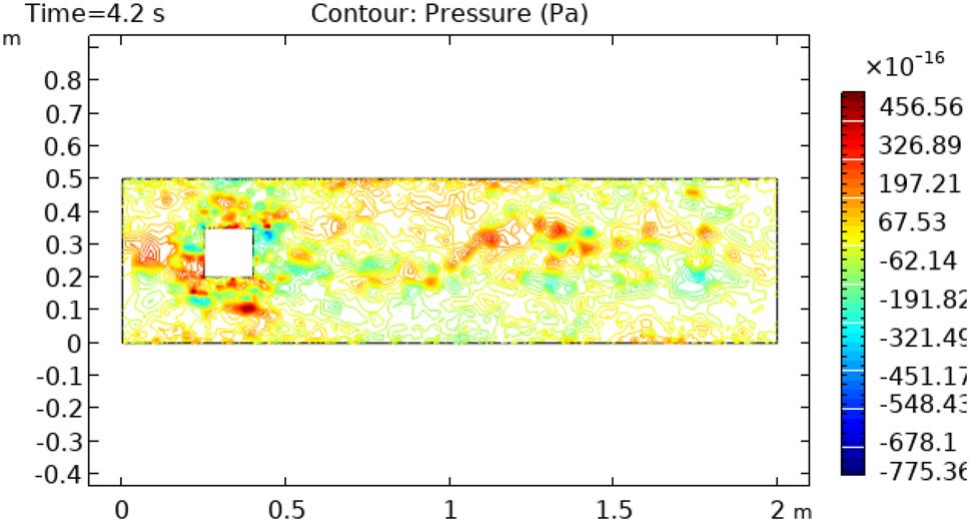
Pressure distribution in squared cylindrical flow at t = 4.2 s.

**Figure 9:**
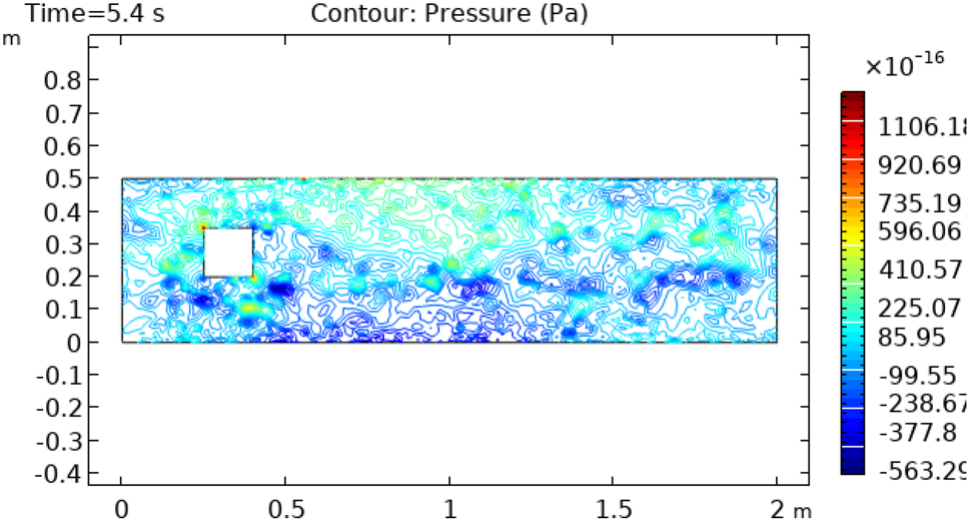
Pressure distribution in squared cylindrical flow at t = 5.4 s.

**Figure 10:**
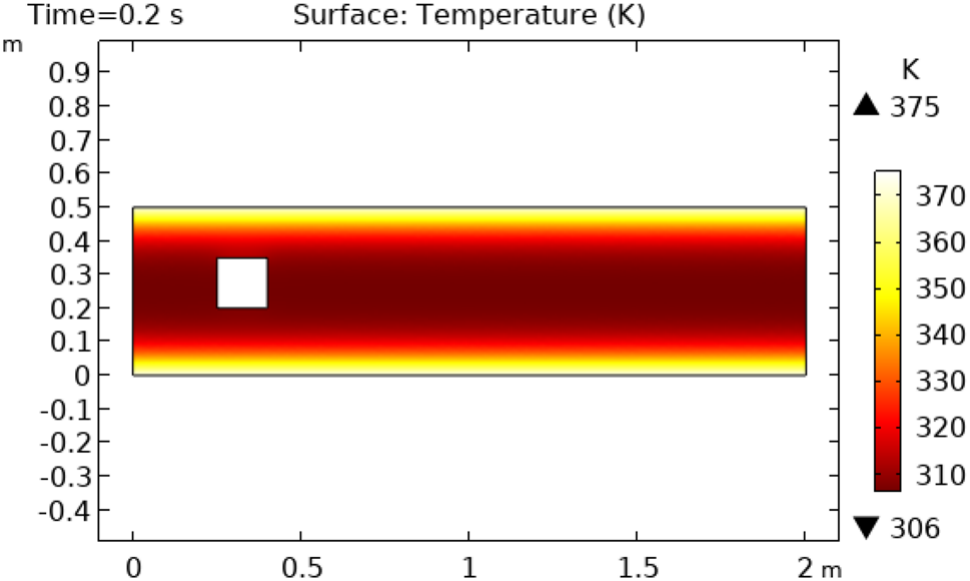
Surface Temperature in squared cylindrical flow at t = 0.2 s.

**Figure 11:**
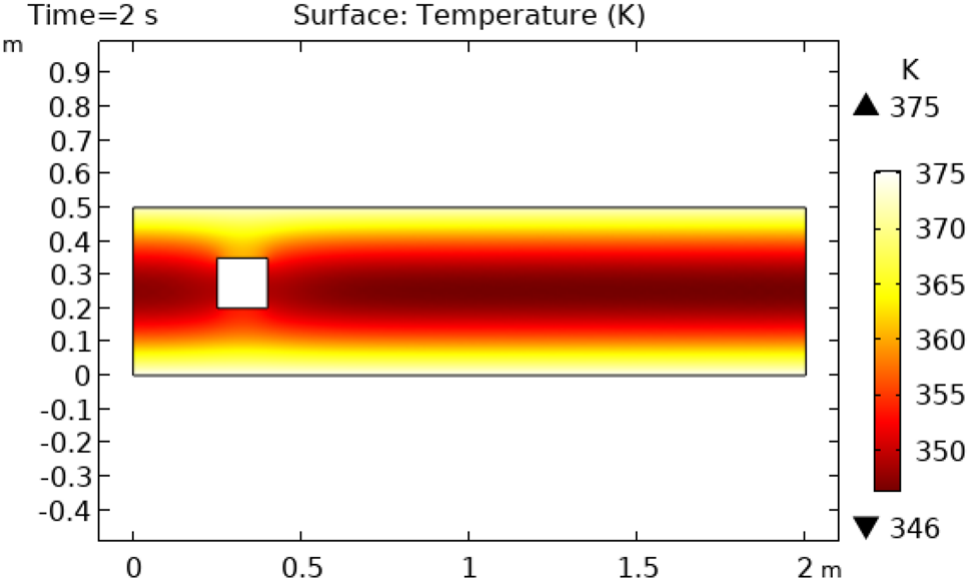
Surface Temperature in squared cylindrical flow at t = 2 s.

Figures 8 and 9 reveal pressure distribution in the flow at the time interval of 4.2 s and 5.4 s, respectively. The value of pressure does not show a regular pattern of variation as it does in previous cases. The range of variation for time interval = 4.2 s is between −775.36 $\times 10^{ - 16} {\text{m}}/{\text{s}}$ and 456.56 $\times 10^{ - 16} {\text{m}}/{\text{s}}$ and that is −553.36 $\times 10^{ - 16} {\text{m}}/{\text{s}}$ and 1106.7 $\times 10^{ - 16} {\text{m}}/{\text{s}}$ for a time interval of 5.4 s. Figure 10 and 11 show the surface temperature around the squared figure block kept in the flow of fluid at time intervals t = 0.2 s and t = 3 s, respectively. Boundary layers of the fluid show the maximum value of temperature throughout the flow which is 360 K in Fig. 10 and 370 K in Fig. 11. The middle layers of flow show minimum values of temperature which is 232 K in Fig. 10 and 280 K in Fig. 11. The other remaining layers oscillate between these maximum and minimum values.

**Figure 12:**
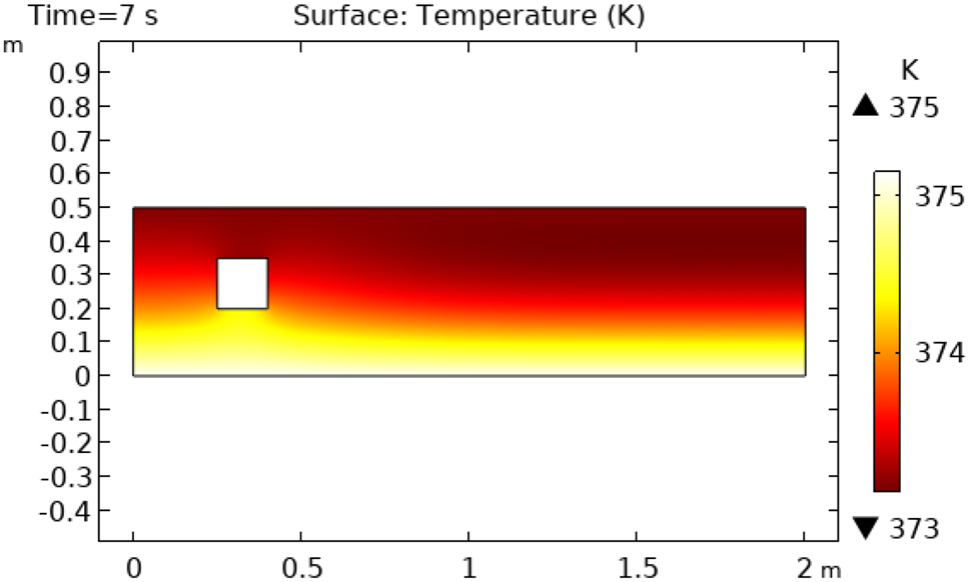
Surface Temperature in squared cylindrical flow at t = 7 s.

**Figure 13:**
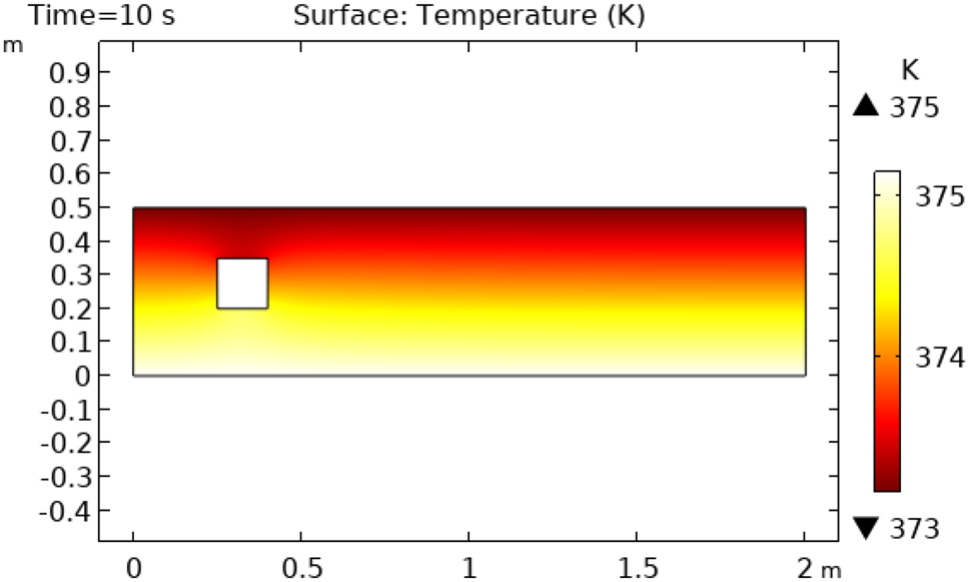
Surface Temperature in squared cylindrical flow at t = 10 s.

**Figure 14:**
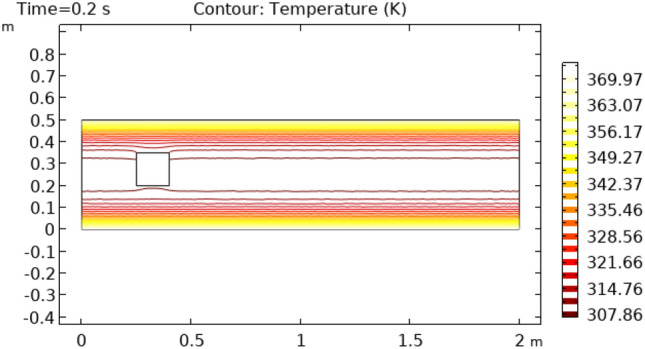
Contour temperature in squared cylindrical flow at t = 2 s.

**Figure 15:**
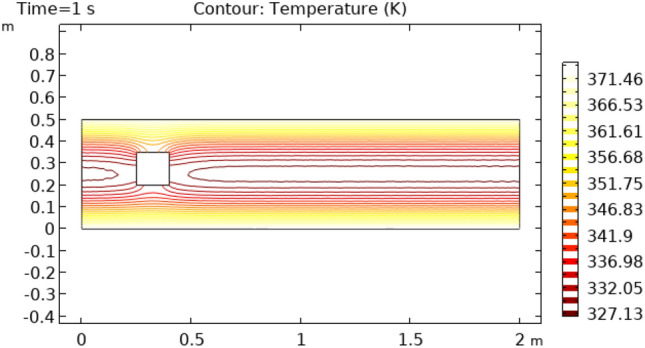
Contour temperature in squared cylindrical flow at t = 5 s.

Figures 12 and 13 describe surface temperature in the outer part of flow at time intervals of 7 s and 10 s respectively. Figures 12 and 13 show approximately the same temperature behavior as Figs. 10 and 11. The difference between these two figures is the maximum and minimum values of temperature. The maximum temperature of Figs. 12 and 13 is 375 K. Minimum temperature in Fig. 12 is 306 K and in Fig. 13 it is 334 K. Figures 14 and 15 show contour temperature around squared block at time intervals of 2 s and 5 s. These figures show that flow possesses minimum values of contour temperature by boundary layers. Minimum contour temperatures are 235.3 K and 282.7 K in Figs. 14 and 15, respectively. The maximum values of contour temperature in Figs. 14 and 15 are 371.56 K and 372.7 K.

**Figure 16:**
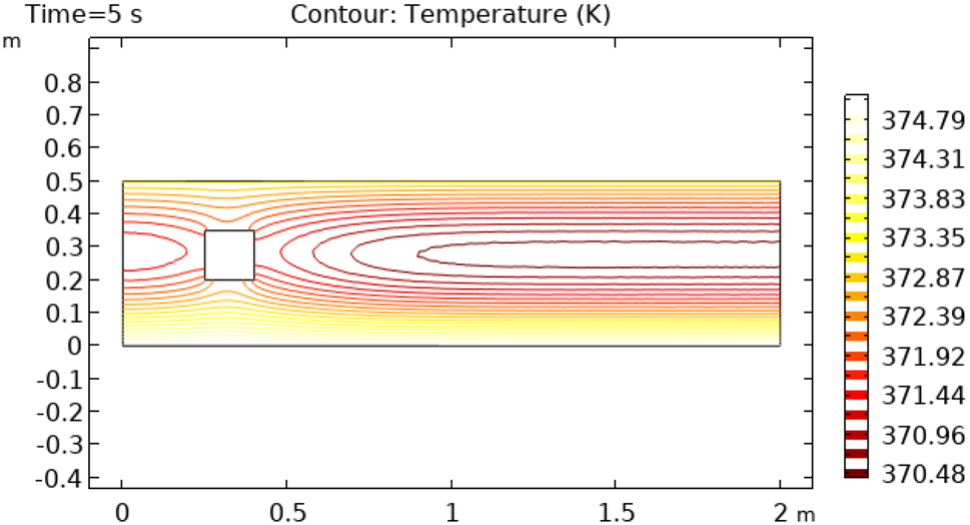
Contour temperature in squared cylindrical flow at t = 7 s.

**Figure 17:**
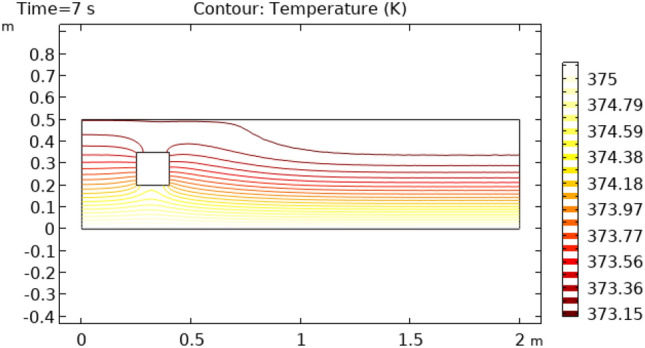
Contour temperature in squared cylindrical flow at t = 10 s.

**Figure 18:**
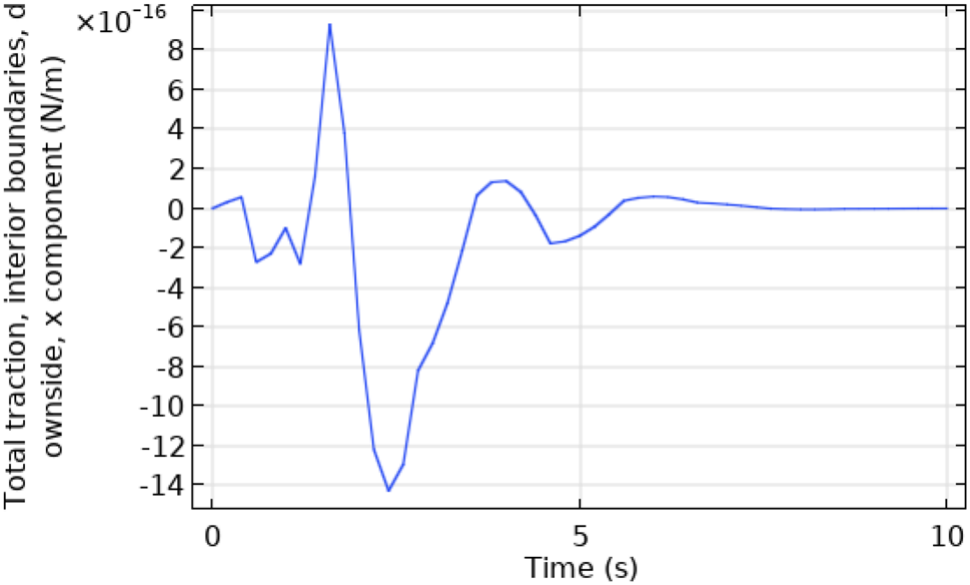
Table graph of drag coefficient in squared cylindrical flow.

Figures 16 and 17 describe the contour temperature on the outer edge of the squared cylinder at times t = 7 s and t = 10 s, respectively. These figures show that boundary layers possess maximum values of contour temperature while middle layers show minimum values of contour temperature. The remaining layers show an oscillation of contour temperature between maximum and minimum values. Maximum values for contour temperature are 373.42 K and 374.12 K in Figs. 16 and 17 respectively. The minimum value of contour temperature in Fig. 16 is 307.72 K and it is 334.45 K in Fig. 17. Figure 18 shows a table graph of the drag coefficient in cylindrical flow. The graph is plotted between time and drag coefficient. This figure shows that with an increase in time, an increase in drag coefficient is also achieved. In a time interval of 10 s drag coefficient increases from 0 to 0.065.

**Figure 19:**
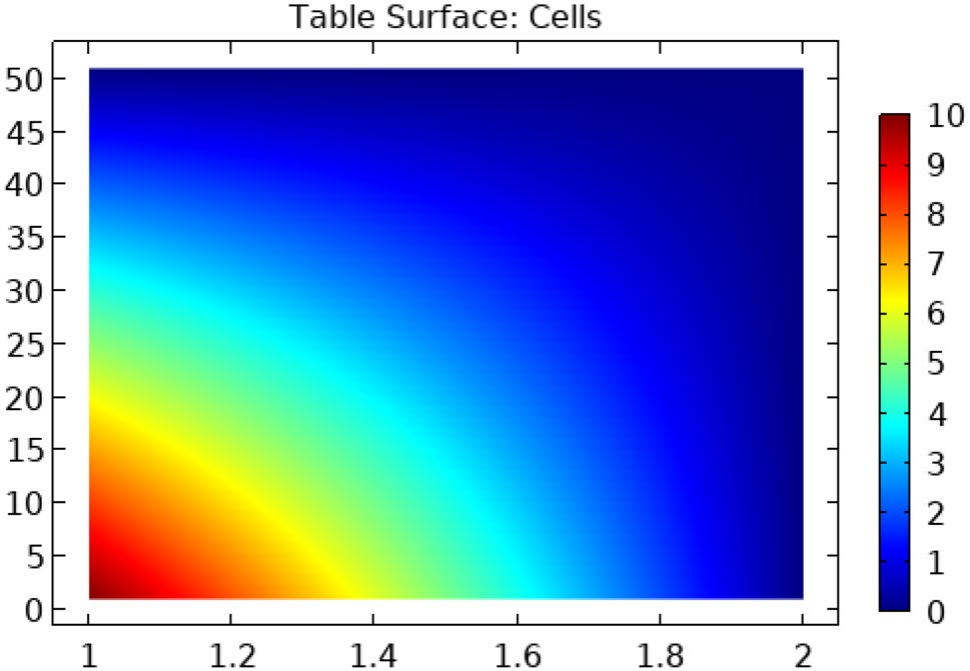
Surface graph of drag coefficient in squared cylindrical flow.

Figure 19 represents a surface graph of the drag coefficient in cylindrical flow. This figure shows that when fluid comes in contact with a squared block then the maximum value of the drag coefficient is produced which ultimately becomes zero. The maximum value of the drag coefficient in this figure is 10.

## Conclusion

The current study highlights the calculated simulation of unsteady two-dimensional fluid flow passing through a squared cylinder. Plots of velocity and pressure distributions are served. Results of heat distribution are also given graphically. The results of the drag and lift coefficients are authorized as well. The flow has a Reynolds number of approximately 150, which is sufficient to maintain the laminar flow. A higher Reynolds number in such a flow causes the laminar flow to be turbulent. A high Reynolds number reduces the drag coefficient as well. When the Mach number is less than 0.3, the flow is compressible. The following are the main findings of the current study:

1. The velocity drops from 2.5 to 1.6 m/s at t = 7 s in the absence of anybody's force and temperature 305.13 K.
2. Pressure increases from 0.00098 to 0.001 Pas in the flow interval of 10 s.
3. Surface temperature increases from 360 to 375 K in time intervals of 10 s keeping pressure constant. And contour temperature increases from 371.56 to 374.2 K in time intervals of 10 s keeping the pressure constant. This information provides us with caution about the emission of steam from the chimneys of furnaces. It implies that when steam flows from a cylindrical geometry like chimneys of furnaces it heats the upper inner and outer parts which may destroy the material. So for safety, that emission should be taken for a short interval of time otherwise it will result in a havoc process.
4. The lift coefficient remains constant and the drag coefficient increases from 0.0005 to 0.065. Under that condition, fluid has to face more resistance. To overcome that difficulty fluid should be provided with high velocity to continue it for a long time.

## Data Availability

The datasets used or analyzed during the current study are available from the corresponding author upon reasonable request.

## Abbreviations

*D*: Diameter of the cylinder (m)
*F*_*D*_: Drag force (kg ms^−^^2^)
*F*_*L*_: Lift force (kg ms^−^^2^)
*p*: Fluid pressure (kg m^−^^1^ s^−^^2^)
*r*: Radical direction (m)
*t*: Time (sec)
*u*: The velocity of the fluid (ms^−^^1^)
$u_{r}$: Radial velocity (ms^−^^1^)
$u_{\theta }$: Tangential velocity (ms^−^^1^)
$C_{p}$: Heat capacity (J/kg K)
$U_{0}$: Normal inflow velocity (m ${\text{s}}^{ - 1} )$
$$U_{mean}$$: Mean velocity (m $${\text{s}}^{ - 1} )$$
*z*: Axial direction
$\rho$: Density of fluid
$\mu$: Reference viscosity of fluid (kg ${\text{ m}}^{ - 1} {\text{s}}^{ - 1} )$
$$\vartheta$$: Kinematic viscosity ($${\text{m}}^{2} {\text{s}}^{ - 1} )$$
$$\nabla$$: Gradient operator ($${\text{m}}^{ - 1} )$$
$\theta$: Tangential direction (rad)
*k*: Thermal conductivity (W/m K)
$\gamma$: Heat capacity ratio
